# Is caregiver burden associated with sex and gender-related characteristics? A large-scale survey study among family caregivers of people with dementia

**DOI:** 10.1186/s12877-025-05795-y

**Published:** 2025-03-14

**Authors:** M. L. A. de Graaff, I. van der Heide, J. J. D. J. M. Rademakers, I. G. M. van Valkengoed, A. L. Francke, A. Woonink, F. M. Bijnsdorp

**Affiliations:** 1 Nivel. Netherlands Institute for Health Services Research, Utrecht, the Netherlands; 2 Department of Family Medicine, School for Public Health and Primary Care (CAPHRI), Maastricht University, Maastricht, The Netherlands; 3 Department of Languages, Literature and Communication, Faculty of Humanities, Utrecht University, Utrecht, The Netherlands; 4 Department of Public and Occupational Health, Amsterdam UMC, Universiteit Van Amsterdam, Amsterdam, the Netherlands; 5 Center of Expertise in Palliative Care, Amsterdam, the Netherlands; 6 Department of Public and Occupational Health, Amsterdam UMC, Vrije Universiteit Amsterdam, Amsterdam, the Netherlands; 7 Alzheimer Netherlands, Amersfoort, the Netherlands; 8 Fyris Research & Data, Den Haag, the Netherlands; 9 Inholland University of Applied Sciences, Amsterdam, the Netherlands

**Keywords:** Dementia, Caregiving, Caregiver, Family care, Informal care, Sex, Gender

## Abstract

**Background:**

The association between the sex of family caregivers and their perceived care burden has been examined thoroughly. The role of sex- and gender-related characteristics of these caregivers in this association remains unknown. We therefore explored the extent to which various gender-related characteristics of caregivers and the sex of people with dementia explain or affect the association between sex of caregivers and their perceived care burden.

**Methods:**

Data were derived from a large-scale survey among Dutch family caregivers of people with dementia in 2022 (*N* = 3067). Both linear and logistic regression analyses were performed to assess mediation of gender-related caregiver characteristics in the association between the sex of the caregiver and the perceived care burden. These characteristics included: hours per week spent on caregiving, being the primary caregiver, relationship with the person with dementia and perceived difficulty in combining daily activities with caregiving. Linear regression analyses were used to assess moderation of the sex of the person with dementia in the association between the sex of the caregiver and the perceived care burden.

**Results:**

Female caregivers perceived a greater care burden than male caregivers. This association was partly explained by female caregivers more often perceiving difficulty of combining daily activities with caregiving than male caregivers. Male caregivers perceived a slightly greater care burden when caring for a female than when caring for a male. The perceived care burden of female caregivers was not related to the sex of the person with dementia.

**Discussion:**

This study highlights how gender-related aspects of family caregiving can contribute to sex differences in perceived care burden. The findings imply that it is important to take gender-related aspects of caregiving into account when developing or offering caregiver support, as support needs differ between male and female caregivers.

**Supplementary Information:**

The online version contains supplementary material available at 10.1186/s12877-025-05795-y.

## Background

Dementia affects not only people with dementia, but also their family caregiver. The caregiving burden is often greater among those who care for a person with dementia, compared to most other disabilities, disorders or illnesses [1]. Recent research has shown that family caregivers of people with dementia, on average, spent over 39 h per week on caregiving and that one in seven family caregivers reported being heavily burdened or overburdened [2]. This burden has been predicted to grow over the next few decades, as people with dementia are becoming increasingly dependent on family care due to the rising costs of professional healthcare and personnel shortages [3, 4]. Family caregivers who feel heavily burdened or overburdened often report stress, anxiety and depression [5, 6].

An increasing number of studies show that female caregivers perceive a greater care burden from providing care to a person with dementia than male caregivers [7–10]. This yields for both physical and mental burden [11, 12]. The higher care burden experienced by female caregivers may be partially attributed to gender-related aspects of caregiving. While *sex* refers to the biological differences between men and women, *gender* encompasses the socially constructed characteristics associated with being male or female, including the norms, behaviours and roles traditionally assigned to each [13].

A key gender-related characteristic of caregiving is that women are more often the primary caregiver than men [14]. Additionally, female caregivers are more likely to care for a parent, sibling, or other relative or friend, whereas male caregivers most often care for their partner [15, 16]. This difference also reflects the fact that female caregivers are more likely than male caregivers to balance family caregiving with other responsibilities, such as employment, social activities, and managing a household [17–19]. On average, male caregivers spend more time on family caregiving than female caregivers [20, 21]. These characteristics are therefore included as gender-related in this study.

As noted above, a vast number of studies have examined the gendered nature of caregiving. However, the extent to which gender-related characteristics of caregiving can function as explanatory variables for the increased care burden among female caregivers compared to male caregivers remains unexplored.

Another understudied aspect of caregiving, is whether the perceived care burden in female and male caregivers is associated with the sex of the person with dementia. Research shows that men and women can display different behaviour depending on the sex of the person they are interacting with [22]. Furthermore, there are indications from previous research that female care recipients are more likely to prefer formal care over informal care, whereas the opposite is true for male care recipients [23]. Notwithstanding, research on same-sex and mixed-sex dyads of caregivers and people with dementia in relation to care burden in family care givers is lacking [24].

Given the aforementioned knowledge gaps, this study aimed to obtain a better understanding of: a) the role gender-related characteristics in the relationship between caregiver sex and care burden, and b) potential differences in care burden between same-sex and mixed sex caregiver-care recipient dyads. The insights gained from this study could inform the development of more tailored support and interventions for male and female caregivers of people with dementia. Accordingly, this study addressed the following research questions:

1. *What is the perceived care burden of family caregivers who cared for a person with dementia in the Netherlands in 2022?*
2. *To what extent does the perceived care burden differ between male and female caregivers?*
3. *To what extent can these differences between male and female caregivers in perceived care burden be explained by gender-related characteristics of the family caregiver?*
4. *To what extent are there any differences in perceived care burden depending on whether a male or female family caregiver cares for a male or female person with dementia?*

## Methods

### Design and sample

We carried out a cross-sectional analysis of data derived from the Dementia Monitor Informal Care 2022 [2]. This concerned a large-scale biennial survey that is conducted by the Netherlands Institute for Health Services Research (Nivel) and Alzheimer Netherlands, the leading Dutch organization for advocacy of people with dementia and their family caregivers. The survey gathered data on the individual characteristics, experiences and opinions of family caregivers of people with dementia. Respondents were eligible if they mentioned caring for a person with dementia, also without a formal diagnosis. Therefore, we did not maintain a strict definition of dementia in this study. Family caregivers were eligible for the survey in 2022 if they were living in the Netherlands at the time and did not provide care as part of their profession. Caregivers did not have to be related to the person with dementia to be eligible. However, the majority of the respondents were related to the person with dementia (98%). For readability, we refer to all respondents as family caregivers. Alzheimer Netherlands recruited the respondents via their extensive network of care professionals, such as case managers. Family caregivers could fill in the survey either online (90%) or on paper (10%). The exact response rate is unknown, as the survey was shared through various social-media channels in an attempt to reach as many caregivers as possible.

The original group of respondents in the Dementia Monitor Informal Care 2022 consisted of 4531 caregivers (N(female) = 3226 & N(male) = 1305). For this paper, we selected 3067 family caregivers based on three criteria. First, the person with dementia had to be alive at the time of the survey, as caregivers often change their perspective on the caregiving process after the death of their person with dementia, with the possible interference of grief [25]. Second, all caregivers had to be caring for a person with dementia who was living in their own home at the time of the survey. Living in a healthcare facility implies receiving professional care daily, which could alter the experience of the family caregiver. Lastly, five caregivers with incomplete data on the items that measured the perceived care burden and the sex of the caregiver and the person with dementia were left out of our analyses, as these items were central to this paper.

### Measurements

#### Perceived care burden

The perceived care burden, conceptualized as the dependent variable, was measured using the following survey question: ‘*How burdened do you feel by providing family care?*’, to be answered on a 5-point Likert scale, ranging from (1) *Not burdened at all* to (5) *Overburdened*. This Likert scale was treated as an interval variable for our analyses. In addition, for descriptive statistics only, the proportion of caregivers who reported feeling heavily burdened or overburdened was determined.

#### Sex

Sex of the caregiver was analyzed as the independent variable in this study. Sex of the caregiver as well as the sex of the person with dementia was self-reported, male or female. To answer the forth research question, sex of the person with dementia was conceptualized as a moderating variable.

#### Gender-related characteristics

Gender-related characteristics were conceptualized as mediating variables in this study. The first gender-related characteristic, caregiving intensity, was measured as total number of hours spent per week on caregiving. Employment status, another gender-related characteristic, was measured as total hours spent on paid work per week. This was categorized as part-time or full-time employed, with the cut-off point for full-time work at 34 h per week. This is in line with the definition of a full-time job, as used by Statistics Netherlands [26]. Caregivers could also report being a student, unemployed or retired, which were then all recategorized as ‘not in employment’. Providing care for a partner as well as ‘being the primary caregiver or not’, was measured dichotomously (yes/no). The extent to which caregivers experienced difficulty in combining caregiving and daily activities was measured using the following answer categories: (1) ‘*not at all*’ or ‘*somewhat*’ and (2) ‘*a lot*’.

#### Covariates

The following background characteristics were also included in the survey and treated in this study as covariates: the age of the caregiver and of the person with dementia, migration background, the number of years since the first symptoms and the perceived difficulty of coping with behavioural changes. The latter item had the following answer categories: (1) ‘*Not at all*’ or ‘*Somewhat*’ and (2) ‘*a lot*’.

The migration background was derived from the country of birth of the caregiver and their parents and was divided into three categories, in line with the categorization used by Statistics Netherlands [27]: (1) ‘*native Dutch background,* (2) ‘*European migration background* and (3) *‘non-European migration background’.*

### Data analyses

To answer the first research question, the mean perceived care burden of family caregivers of people with dementia, as well as the proportion that feels heavily burdened or overburdened was described for both male and female family caregivers (see Table 1). A crude regression analysis displaying the isolated association between sex and care burden was used to answer the second research question (see Table 2, notes, or Table 3 Model 1).

**Table 1:** Descriptive statistics

						Male caregivers (*N* = 906)				Female caregiver (N = 2161)			
						Min.	Max.	Mean or %	SD	Min.	Max.	Mean or %	SD
**Dependent variable**						.	.	.	.	.	.	.	
Mean perceived care burden						0	4	1.6	.9	0	4	1.7	.9
* Heavily burdened (%)*						.	.	9.8	.	.	.	11.7	.
* Overburdened (%)*						.	.	2.9	.	.	.	3.5	.
**Background characteristics**						.	.	.	.	.	.	.	.
Age caregiver						36	94	70.2	11.6	23	97	62.4	11.4
Age of person with dementia						54	98	78.4	7.9	50	100	80.0	8.0
Migration background						.	.	.	.	.	.	.	.
* Native Dutch (%)*						.	.	91.5	.	.	.	91.9	.
* European (excl. the Netherlands) (%)*						.	.	3.9	.	.	.	4.0	.
* Non-European (%)*						.	.	4.6	.	.	.	4.2	.
Difficult to cope with changes in behaviour (%)						.	.	27.2	.	.	.	34.1	.
Years since first symptoms person with dementia						1	22	4.6	3.0	0	25	4.6	2.9
Person with dementia has a formal diagnosis (%)						.	.	92.3	.	.	.	91.1	.
**Gender-related characteristics**						.	.	.	.	.	.	.	.
Mean caregiving intensity (hours per week)						0	168	53.6	56.3	0	168	41.9	53.0
Person with dementia was partner (%)						.	.	69.7	.	.	.	44.2	.
Mean hours of paid work per week						7	100	37.3	10.7	1	68	27.4	9.0
* Parttime (1 – 34 hours) (%)*						.	.	6.2	.	.	.	30.8	.
* Fulltime (>34 hours) (%)*						.	.	17.0	.	.	.	8.9	.
* Unemployed/retired/student (%)*						.	.	73.6	.	.	.	53.0	.
Primary caregiver (%)						.	.	88.1	.	.	.	80.3	.
Difficult to combine daily activities with caring (%)						.	.	13.6	.	.	.	16.4	.
Person with dementia was female (%)						.	.	88.7	.	.	.	40.9	.

**Table 2:** Mediation of gender-related characteristics in the association between sex and perceived care burden

	Effect of female sex on mediator (path a)	Effect of mediator on perceived care burden (path b)	Indirect effect (path ab)	Direct effect (path c’)	BC 95% CI	
*Mediator*					Lower	Upper
Caregiving intensity (hours per week)	−11.635**	.004**	-.025	.061	-.035	-.016
Caring for a partner (ref. = other relative)	−1.067**	.296**	-.044	.042	-.056	-.034
Working parttime (ref. = retired/unemployed)	1.909**	-.143**	-.030	.056	-.046	-.014
Working full time (ref. = retired/unemployed)	-.736**	-.203**	.013	.099	.006	.022
Primary caregiver (ref. = not primary caregiver)	-.594**	.518**	-.033	.053	-.047	-.021
Difficult to combine daily activities with caregiving tasks (ref. = not or somewhat)	.223*	1.091**	.026	.112	.002	.052

*The total effect of (female) sex on perceived care burden was b* = *.086, SE* = *.034, p* < *.05. The outcome of path a for caregiving intensity is the b-coefficient. Other outcomes of path a are log odds. All outcomes of path b are b-coefficients. The indirect effect (path ab) is equal to the difference in the total effect of (female) sex on perceived care burden when correcting for the mediator. The indirect effect is significant when bias-corrected 95% CI does not contain the value 0.000. These confidence intervals are based on 500 resamples*

*p < 0.05

**p < 0.001

**Table 3:** Association between gender-related and background characteristics of caregivers and perceived care burden

			Model 1		Model 2		Model 3		Model 4	
			B	95% CI	B	95% CI	B	95% CI	B	95% CI
Constant			1.617***	1.560—1.674	1.224***	1.146—1.303	.762***	.623—.901	.614***	. 427—.802
Sex of the caregiver (ref. = male)			.086*	.019 – .154	.099**	.025—.173	.074	-.006 -. 153	.256**	.082—.429
Age of the caregiver (centered)					.006***	.004—.009	-.005	-.010—.000	-.005	-.010—.000
Age of the person with dementia (centered)					.002	-.002—.006	.010***	.005—.015	.010***	.005—.015
European migration background (ref. = native Dutch background)					.057	-.110—.225	-.006	-.155—.142	-.007	-.155—.141
Non-European migration background (ref. = native Dutch background)					.098	-.051—.246	.064	-.068—.196	.069	-.063—.201
Difficult to cope with changing behaviour (ref. = not or somewhat)					.632***	. 563—.700	.454***	.392—.516	.457***	.395—.518
Years since symptoms					.041***	. 031—.052	.029***	.020—.038	.029***	.020—.039
Caregiving intensity (hours per week)							.003***	.002—.003	.003***	.002—.003
Caring for a partner (ref. = other relative)							.049	-.081—.179	.017	-.116—.150
Employed part-time (ref. = unemployed or retired)							-.006	-.086—.075	-.005	-.085—.076
Employed fulltime (ref. = unemployed or retired)							-.017	-.121—.086	-.011	-.114—.093
Primary caregiver (ref. = not primary caregiver)							.367***	.286—.448	.365***	.284—.446
Difficult to combine daily activities with caregiving (ref. = not or somewhat)							.892***	.812—.971	.887***	.808—.967
Sex of the person with dementia (ref. = male)							.043	-.028—.113	.231**	.057—.406
Sex of the caregiver * sex of the person with dementia									-.239*	-.442—-.037
Variance Inflation Factor (VIF)					1.05		1.91		3.55	
R^**2**^ (adjusted R^**2**^)			.002 (.002)		.151 (.148)		.347 (.343)		.348 (.344)	

*N (Model 1)* = *3067; N (Model 2)* = *2562; N (Model 3 & 4)* = *2547*

*p < 0.05

**p < 0.01

***p < 0.001

The third research question was approached using mediation analysis, following the four-step method outlined by Baron & Kenny [28], Judd & Kenny [29], and James & Brett [30], with an extension involving the calculation and analysis of the indirect effect based on standardized coefficients. In line with the guidelines proposed by Kenny [31], we defined small, medium, and large effect sizes as 0.01, 0.09, and 0.25 or greater, respectively.

The first step for this analysis was to determine the direct effect of sex on perceived care burden using linear regression analysis (see Table 2, notes or Table 3 Model 1). We then proceeded to analyze the association between sex and each gender-related characteristic using both linear and logistic regression analyses (see Table 2, path a).

The third step of mediation analysis involved calculating the effect of each gender-related characteristic on the perceived care burden using linear regression analyses (see Table 2, path b). The indirect effect was then calculated as the last step of mediation analysis. For this final step, the outcomes of the previous steps (path a & path b) were standardized and then multiplied (see Table 2, path ab). Significance of the indirect effect was calculated by obtaining a 95% confidence interval via bootstrapping.

To answer the fourth and final research question, four consecutive linear regression analyses were conducted (see Table 3). The first model shows the direct effect of sex on the perceived care burden. In the second model, the following background characteristics were added as covariates: age of the caregiver, age of the person with dementia, migration background, experiencing difficulty in to coping with changing behavior and the number of years since the first symptoms of dementia. In the third model the sex of the person with dementia and the following gender-related characteristics were added as covariates: caregiving intensity, caring for a partner, employment status, being the primary caregiver, experiencing difficulty in combining daily activities with caregiving. An interaction term for sex of the family caregiver and the sex of the person with dementia was added in the fourth and last model.

The mean perceived care burden of male and female caregivers caring for a male or female person with dementia could be calculated and then compared by utilizing the standard regression formula [32]. All regression models were tested for multicollinearity using the Variance Inflation Factor (VIF), assuming that a score above 10 indicates strong multicollinearity [33].

Analyses were run only on respondents with complete data for all items in this study. The majority of items contained few, if any, missing values (< 2%). The items that measured the age of the caregiver and the age of the person with dementia contained 7.9% and 10.3% missing values respectively. Meaningful imputation of these missing values was not possible, since the dataset did not contain all relevant general background characteristics, such as level of education, financial situation, or physical health, that could be used for imputation purposes. All statistical analyses were conducted using Stata 16.1 (Stata Corp., TX).

## Results

### Perceived care burden and background characteristics

In our sample, 30% of the respondents were male and 70% were female. We found that 15% of the female caregivers of people with dementia felt heavily burdened or overburdened (see Table 1). This number was slightly lower for male caregivers (13%). Male caregivers were older, on average, than female caregivers (70 years versus 62 years), and male and female people with dementia were of comparable age (78 versus 80). The majority of both male (92%) and female (92%) caregivers did not have a migration background. Experiencing difficulty in coping with changes in the behaviour of the person with dementia was more common among female caregivers than among male caregivers (34% versus 27%). The average number of years since the first symptoms of dementia in the person with dementia was similar for both male and female caregivers (5 years).

On average, male caregivers spent 54 h per week on family care versus 42 h per week in female caregivers. This likely is related to the fact that a greater proportion of male caregivers cared for their partner compared to female caregivers (70% versus 44%). Post-hoc stratification of the average caregiving intensity by caring for a partner or not showed that female caregivers spent more time providing care in both groups (see Appendix, Table 4).

Furthermore, employed female caregivers, on average, had fewer hours of paid work per week than male caregivers (27 versus 37 h). Female caregivers were less likely to be unemployed, retired or a student than male caregivers (53% versus 74%). A greater proportion of female caregivers were employed part-time (31% versus 6%), and a smaller proportion were employed full-time (9% versus 17%). Male caregivers were more likely to be the primary caregiver of the person with dementia than female caregivers (88% versus 80%). Experiencing difficulties in combining daily activities with caregiving tasks was slightly more common among female caregivers than among male caregivers (16% versus 14%).

In addition, 89% of the male caregivers provided care for a female person with dementia and 59% of the female caregivers provided care for a male person with dementia.

### Sex differences in perceived care burden

The crude analysis (with sex as the only independent variable) showed that perceived care burden was greater among female caregivers than among male caregivers (b = 0.086, 95% CI = 0.019—0.154, *p* = 0.012) (see Table 2 notes or Table 3 Model 1). This is the total effect of sex on the perceived care burden and the answer to our second research question.

### Gender-related characteristics as an explanation for differences between sexes in care burden

Our results showed that for all potential mediators or characteristics, a significant association with sex was found. Female caregivers, on average, spent fewer hours per week on caregiving than male caregivers (b = −11.635, *p* < 0.000). Spending more time providing care per week was associated with greater perceived care burden (b = 0.004, *p* < 0.000). The indirect effect of −0.025 was significant, indicating a small negative mediation effect of caregiving intensity in the association between sex and the perceived care burden. In other words, because female caregivers less often provided care for their partner, they appeared to spend fewer hours per week on caregiving.

In addition, female caregivers were less likely to provide care for a partner or spouse with dementia than male caregivers (OR = −1.067, *p* < 0.000). Caring for a partner or spouse was associated with greater perceived care burden (b = 0.296, *p* < 0.000). The indirect effect of −0.044 was significant, which indicates a small negative mediation effect of caring for a partner in the association between sex and the perceived care burden. Post-hoc analysis showed that female caregivers who care for a partner, on average, spent a greater number of hours per week on caregiving than male caregivers who care for a partner (Appendix, Table 4). In addition, female caregivers who do not care for a partner, on average, perceived a greater care burden than male caregivers who do not care for a partner (Appendix, Table 5).

Female caregivers, on average, were more likely to work part-time than male caregivers (OR = 1.909, *p* < 0.000) and working part-time was associated with lower perceived care burden (b = −0.143, *p* < 0.000). The indirect effect of −0.030 was significant, indicating a small negative mediation effect. Female caregivers were less likely to work fulltime (OR = −0.736, *p* < 0.000) than male caregivers and working fulltime was associated with lower perceived care burden (b = −0.203, *p* < 0.000). The indirect effect of 0.013 was significant, indicating a small positive mediation effect. Female caregivers were less likely to be the primary caregiver of the person with dementia (OR = −0.594, p < 0.000) and being the primary caregiver was associated with greater perceived care burden (0.518, *p* < 0.000). The indirect effect of −0.033 was significant, which indicates a small negative mediation effect. Lastly, female caregivers were more likely to experience difficulty in combining daily activities with caregiving (OR = 0.223, *p* = 0.048) and experiencing difficulty in combining daily activities with caregiving was associated with greater perceived care burden (b = 1.091, *p* < 0.000). The indirect effect of 0.026 was significant, indicating a small positive mediation effect.

### The sex of the person with dementia is associated with care burden for male caregivers

The first model in Table 3 showed the crude association between sex and perceived care burden, similarly to the mediation analysis (b = 0.086, 95% CI = 0.019—0.154, *p* = 0.012).

The second model showed that the association between sex and care burden (b = 0.099, 95% CI = 0.025—0.173, *p* = 0.009) persists when accounting for background characteristics. The second model also showed that the age of the caregiver, experiencing difficulty in coping with changing behaviour of the person with dementia and the number of years since the first symptoms of dementia were positively associated with the perceived care burden. The third model showed that after correcting for these variables, the direct association between sex of the caregiver and perceived care burden was no longer present.

The final and fourth model showed that male caregivers perceived a greater care burden in a mixed-sex dyad compared to a same-sex dyad, while the perceived care burden of female caregivers was more similar in a same-sex dyad compared to a mixed-sex dyad (b(interaction) = −0.239, 95% CI = −442—−0.037, *p* = 0.021). On average, the perceived care burden of female caregivers remained greater than the perceived care burden of male caregivers in any dyad. Effect sizes were calculated per dyad: male caregivers caring for a male person with dementia (b = 0.614); male caregivers caring for a female person with dementia (b = 0.845); female caregivers caring for a male person with dementia (b = 0.870); female caregivers caring for a female person with dementia (b = 0.862).

## Discussion

The aim of this paper was to increase our understanding of the differences in perceived care burden between male and female caregivers of people with dementia. Several distinctions between male and female caregivers were highlighted in this paper. Primarily, male caregivers predominantly cared for a female partner with dementia. This is likely linked to men having a lower life expectancy than women in the Netherlands, making men more likely to care for a female partner. Female caregivers relatively often cared for individuals other than their partners. Additionally, male caregivers in our sample more frequently fulfilled the role of primary caregivers, in line with male caregivers relatively often providing care for their partner. Consequently, male caregivers dedicated more time, on average, to caregiving compared to their female counterparts. These distinctions help put our findings in context and offer insight into the differences between male and female caregivers of people with dementia.

Based on the observation that male caregivers, on average, spent more time on caregiving and more frequently cared for a partner with dementia than female caregivers, one might anticipate that male caregivers perceived a greater care burden than female caregivers. This assumption stems from findings from previous studies, which showed an association between caring for a partner and increased caregiving intensity and between caregiving intensity and perceived care burden [24, 34, 35]. However, our findings contrasted this assumption. In response to the first and second research question, 15% of female caregivers of people with dementia felt heavily burdened or overburdened in 2022 compared to 13% of the male caregivers.

Regarding the third research question, our results indicated that gender-related characteristics mediated the association between sex and care burden. Female caregivers are more likely to perceive difficulty in combining daily activities with caregiving tasks, which contributes to female caregivers perceiving a greater care burden. This observation is supported by our finding that female caregivers often combined caregiving with part-time employment and spent more time caring for someone other than their partner (see post-hoc analysis Table 4, Appendix). In contrast, male caregivers appeared to have more clearly defined roles, either as the primary caregiver for a partner or as caregivers dedicating limited time to caregiving while maintaining full-time employment.

These findings highlight the fact that male and female caregivers often have different experiences and are not likely to be in a comparable caregiving situation. Overall, our study’s findings align with previous literature that also found a greater care burden among female caregivers of people with dementia [8, 10, 18, 24]. In addition, our results complement this body of literature by also partially explaining this difference in care burden due to female caregivers being more likely to experience role-conflicts than male caregivers.

The study’s fourth research question addressed whether there is a difference in providing care for a male or female with dementia between male and female caregivers. The results showed that male caregivers perceived a slightly greater care burden when providing care for a female than when providing care for a male. This is likely related to the fact that when male caregivers care for another male (often a father, brother or friend), they are less likely to fulfill the role of primary caregiver compared to male caregivers that take care for a female, which is most often their partner. For female caregivers, the sex of the person with dementia did not affect their perceived care burden, which is likely related to our finding that female caregivers are more intensively involved in taking care for people with dementia other than their partner than male caregivers. However, our findings remained consistent even when account for the relationship with the person with dementia. The results regarding our fourth research question add new knowledge to the body of literature on the impact of interactions between the sex of the caregiver and the sex of the person with dementia as this has not yet been studied before [24].

An important strength of this study is that the recently introduced gender assessment tool, the Stanford Gender-related Variables for Health Research, and the SAGER Guidelines were used as a framework and rationale to study gender-related differences [13, 36].

Another strength of this study is that it was based on large-scale survey data, included various sex- and gender-related characteristics and that it was possible to differentiate between caregiving intensity and perceived care burden. Similar studies were often performed on relatively small sample sizes [7–10].

This study was, however, not without limitations. Caregiving burden has been defined as a multidimensional concept, as physical, emotional, social, financial and psychological stressors are all related to the care burden [8, 24]. Therefore, previous studies have often used multi-item scales, such as the Caregiver Burden Inventory or the Zarit Burden Interview, when analyzing care burden. The single-item measurement of care burden in this study lacks depth and reliability compared to validated multi-item scales. However, as indicated by Pillemer and colleagues (2018), these multi-item scales may not be sensitive to gender due to the fact that male and female caregivers may experience care burden differently. As such, a single-item measurement may allow for more accurate comparisons between male and female caregivers. Future studies are required to validate multi-item scales of care burden in the context of sex and gender research.

Our sample may have been subject to two selection biases. Firstly, respondents who feel heavily burdened or overburdened may not have enough time or energy to fill in an extensive survey. In addition, the respondents in the Dementia Monitor Informal Care 2022 were recruited via a network of care professionals and case managers by Alzheimer Nederland, a leading Dutch organization in the field of dementia research and policy. As such, respondents may have had more knowledge about support options for caregivers than the average caregiver, or may already have received professional support. Both selection biases are more likely to have led to an underestimation of the care burden of caregivers than an overestimation. In reality, the perceived care burden is expected to therefore be greater than is reflected in our results.

## Conclusion

This study showed that on average female caregivers of people with dementia perceived a greater care burden than male caregivers, which can in part be explained by women being more likely to perceive difficulties in combining caregiving tasks with daily activities. These findings highlighted the fact that male and female caregivers often have different experiences and are in different caregiving situation. It implies that, on average, female caregivers might not only need more professional support, but might also more often need different types of support than male caregivers, depending on the situation. For instance, the care burden of a daughter taking care of a parent might be alleviated by receiving support from a case manager or community nurse in care planning and in involving other people in the community in the family care for the parent. A male caregiver taking care of his partner might need support in the form of respite care, for example, in the form of day activity centres for several days per week. Further research on differences in support needs between female and male caregivers of people with dementia, and how their needs best can be met, is therefore recommended. Lastly, it is recommended to include the gender perspective in research on family care in order to develop and offer tailored caregiving support options that help alleviate care burden in caregivers of people with dementia.

## Supplementary Information

Supplementary Material 1.

Supplementary Material 2.

## Data Availability

The datasets used and/or analysed during the current study are available from the corresponding author on reasonable request.
