# Supplementary material for: Is caregiver burden associated with sex and gender-related characteristics? A large-scale survey study among family caregivers of people with dementia
Source: BMC Geriatr. 2025 Mar 14;25:171. doi: 10.1186/s12877-025-05795-y (PMC11908074; doi:10.1186/s12877-025-05795-y)
Supplement: Supplementary file 1 — Supplementary Material 1. [file 12877_2025_5795_MOESM1_ESM.docx]

| **Table 4. Average caregiving intensity in hours per week of male and female caregivers** | | |  |
| --- | --- | --- | --- |
|  | *Female caregiver* | *Male caregiver* | *Difference in means* |
| Person with dementia is not the partner of the caregiver | 13.81 (56%) | 12.53 (30%) | 1.28 |
| Person with dementia is the partner of the caregiver | 77.38 (44%) | 71.90 (70%) | 5.48* |
| ** Difference in mean score between male and female caregivers is significant, based on non-parametric test, p < .05* | | | |

| **Table 5. Average perceived care burden (0-4) of male and female caregivers** | | |  |
| --- | --- | --- | --- |
|  | *Female caregiver* | *Male caregiver* | *Difference in means* |
| Person with dementia is not the partner of the caregiver | 1.61 (56%) | 1.26 (30%) | .35* |
| Person with dementia is the partner of the caregiver | 1.83 (44%) | 1.77 (70%) | .06 |
| ** Difference in mean score between male and female caregivers is significant, based on unpaired t-test, assuming equal variances, p < .05* | | | |
