# Supplementary material for: Is caregiver burden associated with sex and gender-related characteristics? A large-scale survey study among family caregivers of people with dementia
Source: BMC Geriatr. 2025 Mar 14;25:171. doi: 10.1186/s12877-025-05795-y (PMC11908074; doi:10.1186/s12877-025-05795-y)
Supplement: Supplementary file 2 — Supplementary Material 2. [file 12877_2025_5795_MOESM2_ESM.docx]

**Figure 1. Interaction effect of the sex of the person with dementia on the association between sex and care burden of caregiver.**

*Note: X-axis = mean perceived care burden (5-point Likert scale, 0-4)*
